# Stereotactic Radiotherapy for Intramedullary Spinal Arteriovenous Malformations

**DOI:** 10.7759/cureus.2908

**Published:** 2018-07-02

**Authors:** Yoshimasa Mori, Chisa Hashizume, Takahiko Tsugawa, Sachiko Kato, Yuta Shibamoto

**Affiliations:** 1 Radiology and Radiation Oncology, Aichi Medical University, Nagatute, JPN; 2 Nagoya Radiosurgery Center, Nagoya Kyoritsu Hospital, Nagoya, JPN; 3 Radiology and Radiation Oncology, Nagoya City University Gladuate School of Medical Sciences, Nagoya, JPN

**Keywords:** arteriovenous malformation, spinal cord, intramedullary, stereotactic radiotherapy, tetraplegia, paraplegia, embolisation, stereotactic radiosurgery, hemorrhage, venous congestion

## Abstract

Introduction

Preliminary results of stereotactic radiotherapy (SRT) for spinal arteriovenous malformation (AVM) in five cases are presented.

Methods

Two cases were male and three were female. Their median age was 32 years (range: 23 to 54 years). The spinal intramedullary AVMs were located in the cervical spine in three and in the thoracic spine in two. SRT with 20 Gy in four fractions was delivered to the nidus in every case.

Results

At the end of the median follow-up period of 5.5 years (range: 3 to 8.5 years), the neurological symptoms and signs were improved or unchanged as compared with before treatment, although a temporary adverse effect developed between a half year and one year after SRT in one case. The nidi were obliterated five and a half years after SRT in one case and three years after SRT in one. In the other three cases, the nidi were unchanged.

Conclusion

SRT with 20 Gy in four fractions was thought to be a safe treatment, though this study dealt with only a small number of patients with a short-term follow-up period.

## Introduction

Intramedullary spinal cord arteriovenous malformations (AVMs) are rare and challenging to treat. Such lesions usually have multiple feeders that originate from the anterior and/or posterior spinal arteries [1]. Patients present with intramedullary hemorrhage, subarachnoid hemorrhage, or congestive myelopathy [2-3]. The treatment options include embolization and microsurgical resection. However, those are performed effectively only when certain conditions are present, such as favorable location, size, and vascular anatomy allowing management [4-5]. Although it has been reported in many papers that cerebral AVMs can be effectively treated by stereotactic radiosurgery (SRS)/stereotactic radiotherapy (SRT) [6], only a few reports have documented the efficacy of SRS/SRT for spinal intramedullary AVMs [1, 5, 7-10]. Herein, we report our experience of treating five patients with symptomatic intramedullary spinal cord AVM by SRT.

## Materials and methods

This study was approved by the research ethics board of Nagoya Kyoritsu Hospital (K107-02) and by that of Aichi Medical University Hospital (2018-H006). The informed consent was waived.

Five patients with intramedullary spinal AVMs were treated with Novalis® (BrainLAB, Tokyo) SRT at the Nagoya Radiosurgery Center, Nagoya Kyoritsu Hospital from July 2006 through December 2014. Two were male and three were female. Their median age was 32 years (range: 23 to 54 years). The AVMs were located in the cervical (C) spine in three and the thoracic (T) spine in two. Two patients presented with intramedullary hemorrhage causing tetraparesis (Cases 1 and 2). Two patients presented with congestive myelopathy causing tetraparesis (Case 3) and paraparesis (Case 5). One case (Case 4) suffered a hemorrhage and underwent embolization at the initial presentation. Then, five years later, the left leg paresis deteriorated due to congestion myelopathy. In another case (Case 3), embolization was performed prior to SRT. In one case (Case 5), embolization was abandoned due to a positive provocation test.

The true nidus in each case was delineated first on conventional projection angiograms. Next, it was identified as an enhancement volume on computed tomography (CT) angiography. Magnetic resonance imaging (MRI) was not very useful in most cases because it was affected by hemorrhage scars and flow-related changes. SRT with 20 Gy (at the normalization point) in four fractions was delivered to the nidus in every case. Ninety-nine percent of the nidus was covered with 95% dose. Dose selection, 20 Gy in four fractions, was decided in accordance with that reported by Hida et al. [7]. As we wanted to avoid radiation-induced adverse effects on the spinal medulla, a relatively small dose was selected. A Novalis system equipped with both cone collimators and a micro-multileaf collimator (mMLC) with 3-mm thick leaves was used. The method of SRT with the Novalis system has been described previously [11]. The dose planning was done on a BrainSCAN® radiation treatment planning workstation (BrainLAB, Tokyo) in four cases and on an iPlan® (BrainLAB, Tokyo) in one in the latest patients (Case 5). The dose calculation method was the pencil beam method on both workstations.

## Results

Patient characteristics, SRT parameters, and treatment results are shown in Table 1.

**Table 1:** Five Cases Treated by Stereotactic Radiotherapy for Intramedullary Spinal Arteriovenous Malformation Dx: diagnosis; SRT: stereotactic radiotherapy; PTV: planning target volume; FU: follow-up; M: male; F: female; C: cervical; T: thoracic; mos: months; yrs: years; fx: fractions; CR: complete response; NC: no change; MRI: magnetic resonance imaging; CR* (3 yrs): complete response confirmed three years after SRT

Case	Age (yrs)/ Sex	Initial manifestation	Time Dx to SRT	Level	SRT dose PTV volume	FU (yrs)	Nidus	Neurological symptoms	Adverse effects	Survival
1	32 / M	bleeding	11 mos.	C3	20 Gy / 4 fx. 0.1 ml	5.5	CR	improved	(-)	alive
2	54 / F	bleeding	8 mos.	C2-4	20 Gy / 4 fx. 0.7 ml	8.5	CR* (3 yrs）	improved	(-)	alive
3	23 / M	congestion (embolization)	5 yrs.	C3-5	20 Gy / 4 fx. 8.8 ml	3	NC	improved	(-)	alive
4	29 / F	bleeding (embolization) then congestion	5 yrs.	T9-10	20 Gy / 4 fx. 0.3 ml (0.15 + 0.12)	7.5	NC (MRI)	improved	(-)	alive
5	52 / F	congestion	18 mos.	T10-11	20 Gy / 4fx. 3.2 ml	3	NC (MRI)	stable	temporary	alive

At the end of the median follow-up period of 5.5 years (range: 3 to 8.5 years), the neurological symptoms and signs were improved or unchanged in all five cases as compared with before treatment, although a temporary adverse effect developed between a half year and one year after SRT in one (Case 5). No hemorrhage occurred in any patient during the follow-up period. The nidi were obliterated five and a half years (Case 1) and three years (Case 2) after SRT in two of the five cases. In the other three cases, the nidi were unchanged on angiography or MRI.

Case 1

**Figure 1:**
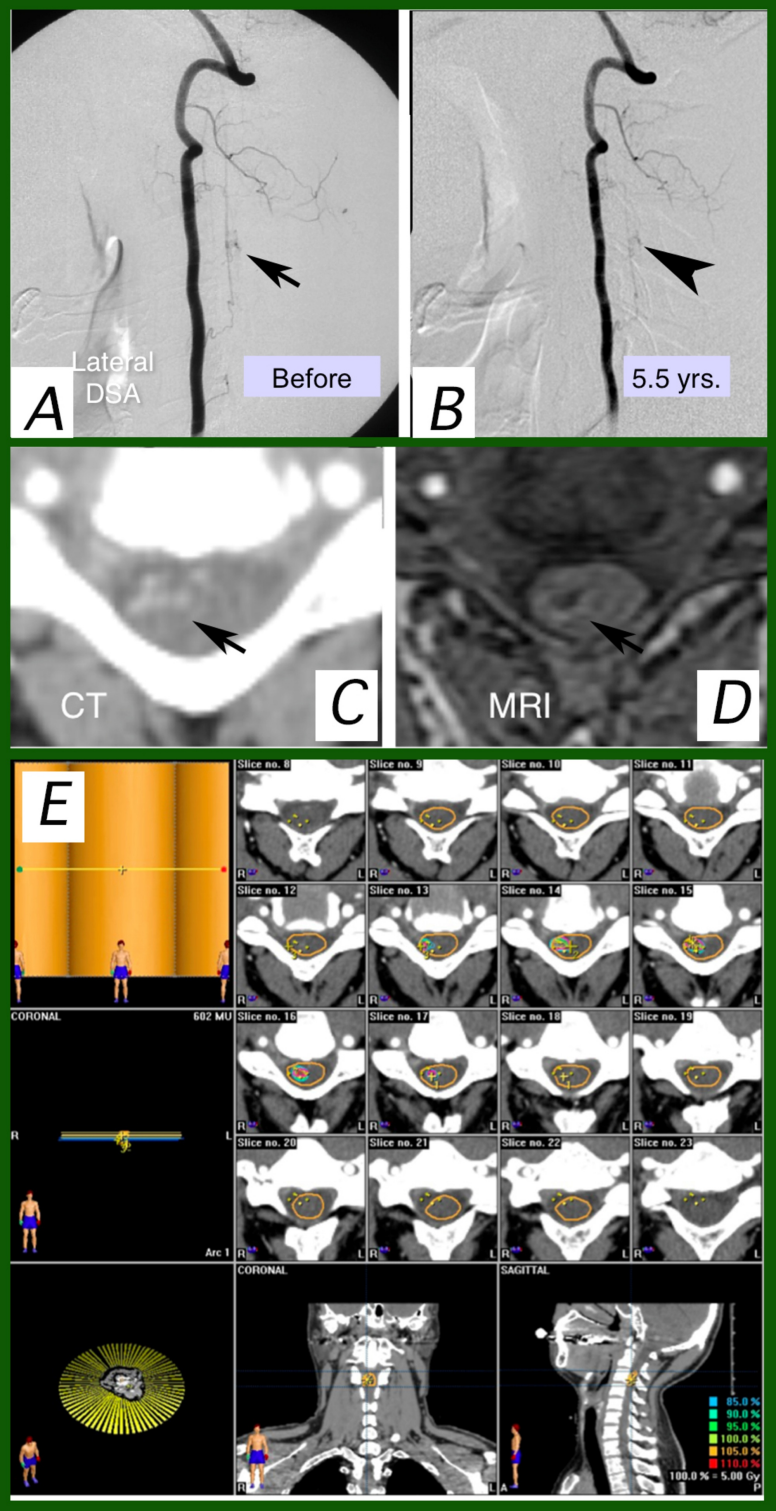
Case 1 1A: Lateral views of right vertebral angiography before and (1B) five and a half years after stereotactic radiotherapy (SRT). 1A: small nidus fed by the anterior spinal artery and draining posteriorly (arrow); 1B: arteriovenous shunt flow disappeared (arrowhead), though the dilated vessel located posteriorly was still observed; 1C: the nidus was located on the right side of the spinal medulla as a contrast-enhanced area (arrow) with vessels on pre-SRT CT; 1D: pre-SRT gadolinium-enhancement spoiled gradient echo MRI was not very useful to delineate the nidus because low-intensity hemosiderin affected the surrounding findings (arrow); 1E: dose-planning of SRT on BrainSCAN workstation. A three-arc cone-collimator plan was employed. CT: computed tomography; MRI: magnetic resonance imaging; SRT: stereotactic radiotherapy

A 32-year-old male was diagnosed as having a C3 AVM (Figure 1). Intramedullary hemorrhage once caused tetraplegia. SRT was done 11 months later in the chronic state after the tetraparesis was largely improved by conservative therapy. No adverse effects were observed after SRT. Angiography performed five and a half years later disclosed complete nidus obliteration.

Case 2

**Figure 2:**
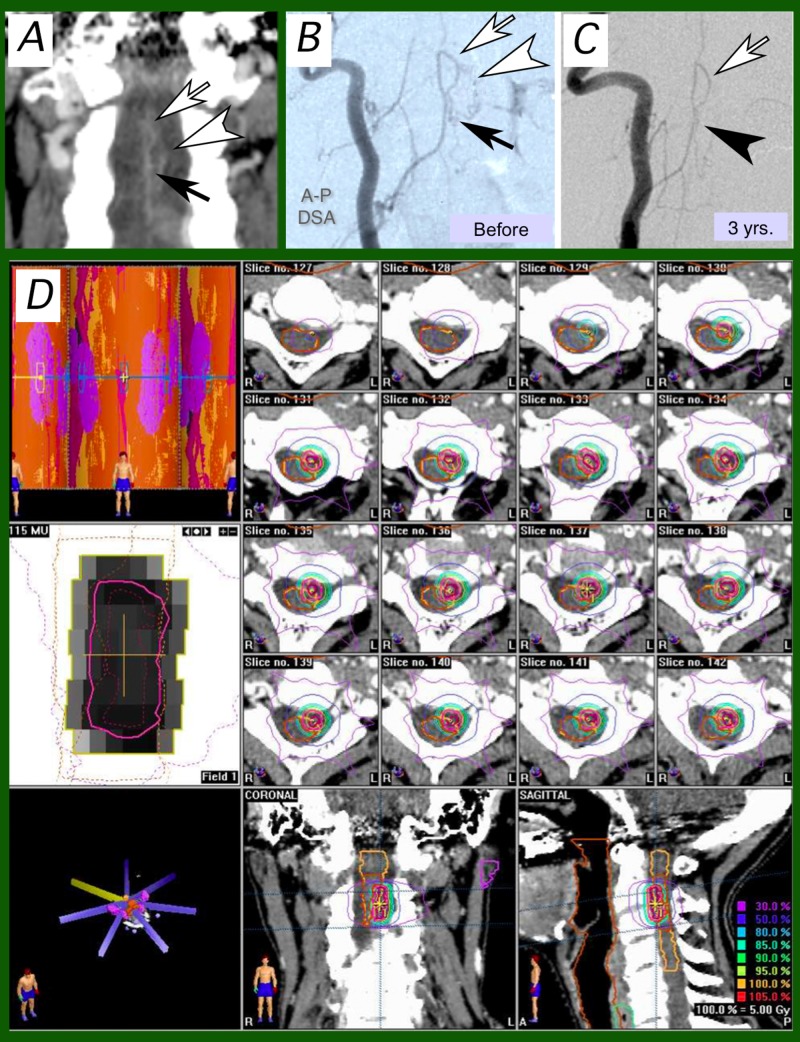
Case 2 2A: Pre-stereotactic radiotherapy (SRT) coronal image of CT and (2B) frontal views of the right vertebral angiography before and (2C) three years after SRT. A little longitudinally long and narrow nidus (arrows, 2A, B) was disclosed and a proximal artery loop (white arrows, 2A-C) and draining veins (white arrowheads, 2A, B) connected to the venous plexus was observed on pre-SRT angiography (2B). 2C: the nidus disappeared, and early venous filling was not observed (arrowhead) three years after SRT; 2D: dose-planning of SRT on BrainSCAN workstation. Seven-beam intensity modulated-SRT plan was employed. CT: computed tomography

A 54-year-old female was diagnosed as having a C2-C4 AVM (Figure 2). Hemorrhage once caused tetraplegia. SRT was done eight months later in the chronic state after the tetraparesis was largely improved by conservative therapy. No adverse effects were observed after SRT. Angiography performed three years later disclosed complete nidus obliteration.

Case 3

**Figure 3:**
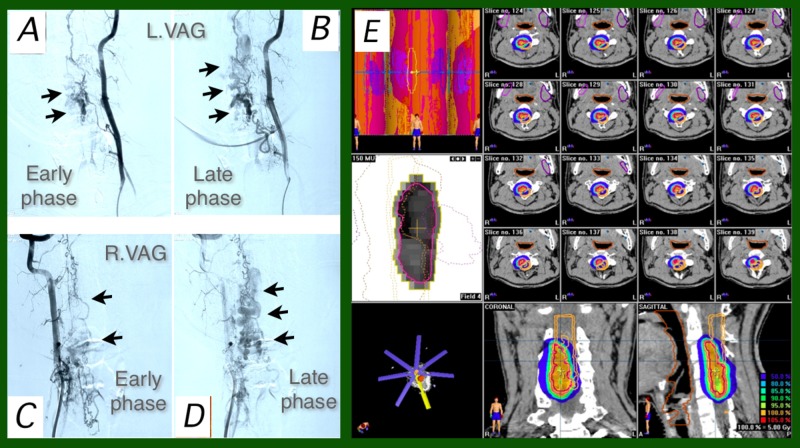
Case 3 Frontal views of pre-stereotactic radiotherapy (SRT) post-embolization (three sessions) left vertebral angiography (3A-B) and right vertebral angiography (3C-D). A massive nidus (arrows) was disclosed with multiple feeding arteries from both sides and tortuous draining veins; 3E: dose-planning of SRT on BrainSCAN workstation. Seven-beam intensity modulated-SRT plan was employed.

A 23-year-old male was diagnosed as having a C3-C5 AVM (Figure 3). A gradual deterioration of tetraparesis developed during the five-year period after the diagnosis of the spinal AVM. SRT was done after incomplete improvement of symptoms by three-time embolization performed during a one-month period. The intramedullary nidus was embolized only partially, but his neurological symptoms started to improve after the embolization procedures. After the SRT, the patient’s neurological symptoms continued improving from wheelchair to walking and then running.

Case 4

**Figure 4:**
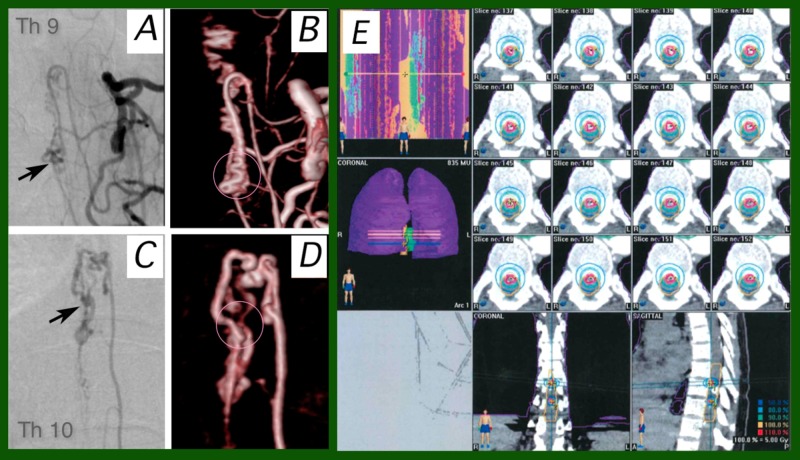
Case 4 Frontal views of left spinal angiography (4A, C) and CT angiography (4B, D) five years after embolization following hemorrhage. Two portions of the residual nidus (arrows) were identified at the levels of T9 (4A, B) and T10 (4C, D). 4E: dose-planning of stereotactic radiotherapy (SRT) on BrainSCAN workstation. Two-arc SRT plan, with one arc each for each portion of the nidus, was adopted. CT: computed tomography; T: thoracic

A 29-year-old female was diagnosed as having a T9-10 AVM (Figure 4). A hemorrhage causing right leg paresis occurred initially. Incomplete embolization of an intramedullary spinal AVM was performed. After a five-year stable period, a second deterioration of symptoms occurred. Left leg paresis was added to the right leg paresis. SRT was performed after a two-month stable period. No adverse effects were observed.

Case 5

**Figure 5:**
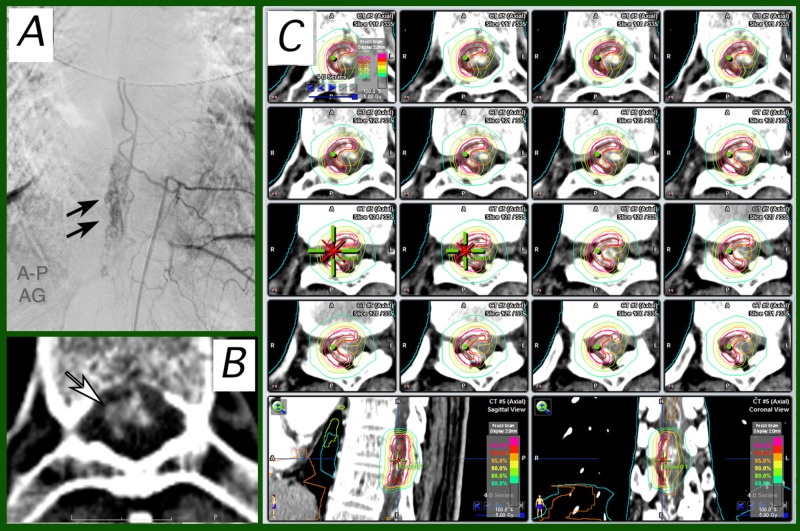
Case 5 Frontal view of left spinal angiography (5A) and axial CT (5B) before stereotactic radiotherapy (SRT). A little longitudinally long and narrow nidus was demonstrated (arrows, 5A). The nidus and draining veins were revealed as an irregular-shaped contrast-enhancement area in the spinal canal (white arrow, 5B) on CT. As a provocation test worsened the neurological signs, embolization was not performed. 5C: dose-planning of SRT on the iPlan workstation. Seven-beam intensity-modulated SRT plan was employed.

A 52-year-old female was diagnosed as having a T10-11 AVM (Figure 5). A gradual deterioration of the paraparesis developed. Embolization was abandoned because of a positive provocation test. SRT was done 18 months later. Around six months later, she developed a temporary slight deterioration of the paraparesis which was thought to represent radiation-induced adverse effects or a fluctuation of symptoms due to congestive myelopathy. A little temporary spread of T2 high-intensity change was observed on MRI. 

## Discussion

Intramedullary AVM has a nidus in the parenchyma of the spinal medulla, which is similar to that of an intracranial AVM. The first-line treatment is embolization because surgical treatment aiming to remove the nidus from the spinal cord carries a significant intraprocedural risk. Surgery should only be indicated for symptomatic cases when embolization is too hazardous or if endovascular treatment results in an incomplete obliteration [3]. Complete angiographical obliteration of the nidus is not necessarily the goal of embolization, but rather the treatment aims to reduce the shunting volume and stabilize symptoms. Furthermore, both complete and partial endovascular treatments have been shown to significantly decrease the risks of future hemorrhage [3, 12]. SRS/SRT is another possible treatment option for intramedullary spinal AVM, though only a few series have been reported thus far [1, 5, 7, 9-10]. The reported series are summarized in Table 2.

**Table 2:** Reported Series of Stereotactic Radiosurgery / Stereotactic Radiotherapy for Intramedullary Spinal Arteriovenous Malformations No: number; pts: patients; fx: fractions; FU: follow-up; C: cervical; T: thoracic; NA: not available; RTRT: real-time tumor-tracking radiotherapy; CK: CyberKnife; AG: angiography; MRI: magnetic resonance imaging; PR: partial response (nidus diminished); CR: complete response (nidus obliteration)

Authors (Year)	No. of pts.	Spinal level (No. of cases)	Nidus volume (ml)	Modality	Dose (Gy / fx.)	FU (mos.)	Outcomes	Adverse effects
Hida et al. (2003) [7]	10	C: 4 T: 6	N.A.	RTRT	20-40 / 4-20	26 – 124 (mean 49)	PR on AG and MRI in 5 of 7 with AG FU	none
Potharaju et al. (2014) [10]	3	T: 3	2.96 – 4.93	CK	21 / 3	24 – 39 (mean 36)	PR on MRI in 2 of 2 with imaging FU	none
Kalani et al. (2016) [9]	37	C: 19 T: 12 Conus: 6	0.2 – 1.5 (mean 2.3)	CK	20 - 21 / 2 - 3	2 – 121 (mean 39.7)	CR in 19% (7/37); PR in 27% (10/37)	myelopathy in 3% (1/37)
Rashad et al. (2017) [1]	5	C: 2 T: 3	NA	CK	18 / 3	16 – 62 (mean 37.2)	PR in 1 of 5	dysesthesia increase in 1 of 5
Present study (2018)	5	C: 2 T: 3	0.1 – 8.8	Novalis	20 / 4	3 – 8.5 yrs.	CR in 2 of 5	temporary in 1 of 5

Initially, Hida et al. [7] and Sinclair et al. [5] published their experience in SRS/SRT for spinal AVMs. Hida et al. reported their experience with a linear accelerator system. They treated 10 patients with hypofractionated linear accelerator SRT, all of whom presented with hemorrhage. However, no new hemorrhages were observed during the follow-up period. Although no angiographical cure of the AVM was observed, five patients attained improved motor functions. Sinclair et al. reported a series of 15 patients treated by the CyberKnife® (Accuray, Inc., Sunnyvale, CA, USA). In addition, Adler et al. [8] and then Kalani et al. [9] renewed their data on the CyberKnife for spinal AVMs at Stanford University. Kalani et al. [9] recently reported treatment results in 37 patients, which is the largest population report on spinal AVM SRS/SRT in the literature. The treatment dose was increased gradually. The first patients were treated with 20 Gy, delivered in three to four sessions. Given the lack of observed morbidity in the initial groups, the dosage was increased to 21 Gy, delivered over three sessions. Their current protocol delivers 20 Gy over two sessions. This dose of 10 Gy per session to the surrounding spinal cord is tolerable in most patients. In addition, two patients with very small conus-region spinal cord AVMs were treated with a single session of SRS. The mean follow-up period for the study was 39.7 months (2 - 121 months). There was a total AVM obliteration rate of 19% (7/37) and a significant reduction rate in an additional 10 cases (27%) among the patients who had undergone formal spinal angiography at the three-year follow-up.

Potharaju et al. [10] reported the results in three cases. They delivered 21 Gy in three fractions by the CyberKnife. In their experience, two of the three patients experienced significant symptomatic improvement. One patient did not have any prior intervention, whereas the other had embolization done twice before undergoing SRT. Postprocedure MRI revealed an absence of flow voids, suggesting a good radiologic response in these two patients. The third patient also had embolization done twice. He did not show any clinical improvement, probably because he had irreversible damage to the spinal cord. Rashad et al. [1] reported the results in five cases, including one case of a spinal arteriovenous metameric syndrome. They used a lower radiation dose (biological equivalent dose (BED) = 72 Gy) and delivered 18 Gy in three fractions using the CyberKnife. 

The dose delivered to successfully obliterate an intracranial AVM nidus varies between 14 and 24 Gy in a single fraction, depending on the location and size of the AVM. A similar dose to the spinal cord in a single fraction may result in radiation myelitis. Radiation myelitis is irreversible and hence limits what dose can be safely delivered to the spinal cord. The normal spinal cord tolerance to radiotherapy is considered to be 45 to 50 Gy in 23 to 25 fractions across five weeks. However, in a radiosurgical setting, the radiotolerance doses are less well-established. In our study, a relatively low dose, the same as that reported by Hida et al. [7] and Rashad et al. [1], was used. None of the patients demonstrated evidence of hemorrhage or persistent neurological deterioration attributable to SRT. Furthermore, in the whole cases of reported series, including ours, no hemorrhage was observed during the follow-up period (Table 2). Hemorrhage might be prevented after SRT even when the nidus is not completely obliterated [8]. 

In two of five patients, each of whom had a small nidus (0.1 ml and 0.7 ml), successful complete obliteration of the nidus was obtained in our series. A low dose might be enough to treat a small nidus. However, in the series of Hida et al. [7] and Potharaju et al. [10], no case achieved complete obliteration of the nidus. Kalani et al. [9], who used a greater dose, stated that their obliteration rate of 19% did not correlate with lesion volume. One of the highest degrees of AVM obliteration was in a lesion measuring 15 ml. In addition, Rashad et al. [1] presented no case with complete obliteration of the nidus either. Spinal cord AVMs are heterogeneous and lie along a spectrum of conditions involving aberrant blood vessels. The limitations of our report include a small number of patients. More clinical experience and longer follow-up are required to decide on the optimal dosage, fractionation, and recognition of late side effects of SRS/SRT.

## Conclusions

Our data are encouraging and support SRT as a new option for treating spinal AVM, particularly when patients are not amenable to surgical or endovascular obliteration. SRT with 20 Gy in four fractions was thought to be a safe treatment, though this conclusion was based on only a small number of patients. Long-term follow-up will be required to validate the safety of SRT for the management of spinal cord AVMs.
